# Monitoring the Cure State of Thermosetting Resins by Ultrasound

**DOI:** 10.3390/ma6093783

**Published:** 2013-09-05

**Authors:** Francesca Lionetto, Alfonso Maffezzoli

**Affiliations:** Department of Engineering for Innovation, University of Salento, Via per Monteroni, 73100 Lecce, Italy; E-Mail: alfonso.maffezzoli@unisalento.it

**Keywords:** ultrasonic wave propagation, ultrasonic dynamic mechanical analysis, viscoelastic properties, cure monitoring, gelation, vitrification, longitudinal modulus, thermosetting resins

## Abstract

The propagation of low intensity ultrasound in a curing resin, acting as a high frequency oscillatory excitation, has been recently proposed as an ultrasonic dynamic mechanical analysis (UDMA) for cure monitoring. The technique measures sound velocity and attenuation, which are very sensitive to changes in the viscoelastic characteristics of the curing resin, since the velocity is related to the resin storage modulus and density, while the attenuation is related to the energy dissipation and scattering in the curing resin. The paper reviews the results obtained by the authors’ research group in the last decade by means of in-house made ultrasonic set-ups for both contact and air-coupled ultrasonic experiments. The basics of the ultrasonic wave propagation in polymers and examples of measurements of the time-evolution of ultrasonic longitudinal modulus and chemical conversion of different thermosetting resins are presented. The effect of temperature on the cure kinetics, the comparison with rheological, low frequency dynamic mechanical and calorimetric results, and the correlation between ultrasonic modulus and crosslinking density will be also discussed. The paper highlights the reliability of ultrasonic wave propagation for monitoring the physical changes taking place during curing and the potential for online monitoring during polymer and polymer matrix composite processing.

## 1. Introduction

Thermosetting resins are gaining increasing use in many structural composite applications in different fields and in the developments of polymer nanocomposites [1,2,3]. The main peculiarity of thermosets is that, during their processing, they undergo a molecular crosslinking process, called “cure”, during which they change irreversibly from being viscous liquids to rigid and highly cross-linked polymer solids. The ability to process thermoset composites and nanocomposites into useful forms depend upon controlling the interplay of the thermoset composition (e.g., resin, curing agent, nanoparticles, *etc*.) and its effect on the state of cure (onset of the crosslinking reaction, matrix gelation, rate of cure reaction).

There is a growing interest in methods to track the cure reaction and to assess the post-cure properties of the formed component. Cure monitoring is of the utmost importance in order (i) to provide necessary conditions for proper epoxy wetting or impregnation; (ii) to distinguish the different stages of the structural formation in order to define the process time window; and (iii) to be able to identify the degree of cure that satisfies the minimum requirements and the optimum cure cycles of a matrix resin [4]. Cure monitoring is, thus, an important tool for ensuring manufacturing reliability and reproducibility of composite parts. At last, but not least, cure monitoring presents the potential for the future automation of composite manufacturing.

Over the past decades, different techniques have been applied to cure monitoring of thermosetting resins, including differential scanning calorimetry (DSC) [5,6], dynamic mechanical thermal analysis (DMTA) [7,8], dielectric analysis (DEA) [8,9,10], Fourier Transform FT-IR spectroscopy [11], near infrared spectroscopy (NIR) [12,13], and Raman spectroscopy [14]. However, many of these cure monitoring methods are laboratory based and not suitable for industrial application, require expensive equipment, are destructive, and typically are suited to working with small samples of restricted dimensions [15].

Recently, ultrasonic dynamic analysis (UDMA), based on the propagation of ultrasonic waves, has been proposed by the authors as a high frequency extension of current dynamic mechanical analysis methods [16]. The application of ultrasonic waves to a material, in fact, acting as a high frequency oscillatory excitation, provides information regarding the viscoelastic behavior of polymers. Due to the high frequency and small amplitude of the periodic oscillations applied to the sample, ultrasonic dynamic mechanical analysis probes the small-scale mobility of short chain segments, which generally does not involve the entanglements [17].

Ultrasonic cure monitoring is based on the measurement of velocity and attenuation of ultrasonic waves, which are very sensitive to changes in the viscoelastic characteristics of forming macromolecular networks. The speed of sound in a material is dependent on its density and modulus, thus, directly reflecting the state of the cure and giving an immediate indication of the increase of material mechanical stiffness [18]. The major advantages of ultrasonic cure monitoring are related to (i) its non-destructive character; (ii) the possibility of monitoring with high sensitivity the overall curing process; and (iii) the potential to be used on-line and *in situ* during processing of composite materials for predicting their final properties.

Despite its peculiar advantages, the application of ultrasonic cure monitoring has been limited in the past decades by the inadequate long-term stability of transducers at high temperatures, the lack of commercial ultrasonic instrumentation, and by the requirement of a fluid coupling medium between the ultrasonic transducer and the material under testing. The presence of a coupling medium can cause large transit time errors, change the waveform and, hence, affects the accuracy of the velocity measurement. Researchers usually construct their own set-up from commercially available components (piezoelectric crystals, electronic equipment, *etc.*), which require a fairly good understanding of the underlying principles [19,20,21,22,23].

In this paper, a review of the recent experimental research carried out by the authors on ultrasonic cure monitoring will be used to illustrate the reliability and potential of ultrasonic wave propagation for online monitoring of curing during polymer and composite processing. In the first part, the principles of ultrasonic dynamic mechanical analysis of polymers will be summarized. Then, the details concerning the in-house build experimental set-up for contact and air-coupled ultrasonic cure monitoring will be presented together with the major results obtained by the authors over the past decade on unsaturated polyester and epoxy resins.

## 2. Basis of Ultrasonic Wave Propagation in Polymers

Ultrasonic waves are mechanical vibrations (in the region of 20 kHz–100 MHz), which propagate through very small displacements of atoms and chain segments around their equilibrium positions. In the case of polymers, the forces acting along chain segments and between molecular chains, create displacements into neighboring zones, thus, creating stress waves through the material [24].

Several kinds of ultrasonic waves may propagate through solids, namely longitudinal waves, shear waves, Rayleigh waves (or surface acoustic waves), and Lamb waves (or plate waves). In longitudinal waves, the material is subjected to alternate local compression and expansions and the motion of the particles of material transmitting these waves is in the same direction as the propagation of the wave. In shear waves the solid is locally subjected to shearing forces and the particle motion is perpendicular to the direction of the propagation of the wave [25]. Since gases and liquids are practically incapable of transmitting shear, for ultrasonic cure monitoring, longitudinal waves are normally preferred to shear waves, which present a very high level of attenuation in liquids and soft gel samples [25]. However, shear waves can be used to follow the curing process after the gelation stage [22,26].

Ultrasonic waves are characterized by a wavelength, amplitude of displacement, and velocity of propagation. In most applications, ultrasonic waves are generated with a transducer, which converts electrical energy into ultrasonic waves. The same transducer (or a second one) will convert the ultrasonic wave back to an electrical signal for further analysis [27].

The acoustic characteristics of a material are determined by two parameters, the ultrasonic velocity, *c*; and the ultrasonic attenuation coefficient, *a*. The first is the velocity of propagation of elastic waves, which is calculated from the measured “time of flight”, that is the time taken by the sound to propagate through the sample. The speed of sound in a homogenous medium is directly related to both elastic modulus and density; thus, changes in either elasticity or density will affect pulse transit time through a sample of a given thickness.

The attenuation is a measure of dissipative energy, converted to heat, as the wave propagates through the material. In addition to reflection losses at macroscopic defects or other material discontinuities, most of the energy loss results from the absorption and the scattering of ultrasonic waves. The scattering contribution is considerable when the medium is structurally heterogeneous and contains particles of size comparable to the wavelength of the propagating waves, as in the case of filled polymers and some crystalline polymers. The extent of energy absorption is related to molecular rearrangements in the polymer structure, such as glass transition, melting, secondary transitions, and to chemical reactions such as those occurring during curing of thermosetting resins [25,28]. The attenuation coefficient is determined from the change in the amplitude of the acoustic signal.

In order to have an accurate assessment of the sound velocity and attenuation in a material, it is necessary the measurement of the specimen thickness and its variations eventually occurring during the test.

The acoustic response of a polymer can provide information about viscoelastic behavior, because it can be directly used to calculate the two components of the complex modulus. As mentioned earlier, the use of longitudinal waves is preferred to shear waves to establish more controllable and measurable attenuation conditions. The propagation of longitudinal elastic waves can, in fact, be tracked even in liquid mixtures of monomers or oligomers.

When the sample dimension normal to the direction of the acoustic wave propagation is large compared to the wavelength, the measurement of ultrasonic velocity and attenuation may be used to calculate the storage (*L*′) and loss (*L*″) components of the longitudinal modulus from the following expressions [28]:

(1) $$L\prime =\frac{\rho c^{2}\left[1-{\left(\frac{a\lambda }{2\pi }\right)}^{2}\right]}{{\left[1+{\left(\frac{a\lambda }{2\pi }\right)}^{2}\right]}^{2}}\text{ and }L\prime \prime =\frac{2\rho c^{2}\left(\frac{a\lambda }{2\pi }\right)}{{\left[1+{\left(\frac{a\lambda }{2\pi }\right)}^{2}\right]}^{2}}$$

where ρ is the material density; *c* the ultrasonic velocity; *a* the ultrasonic attenuation coefficient; and λ is the wavelength of propagating waves, obtained from the ratio of velocity to frequency *f*, (λ= *c*/*f*). In linear elasticity, the longitudinal modulus is one of the elastic moduli available to describe isotropic homogeneous materials. *L*′ and *L*″ are related to the bulk (*K*′ and *K*″) and shear (*G*′ and *G*″) moduli from the equations [21,25,28,29,30]:

*L*′ = *K*′ + 4/3 *G*′ (2)

*L*″ = *K*″ + 4/3 *G*″ (3)

Note that these relationships are valid for plane strain conditions, where *L*′ corresponds to the elastic modulus for specimens where the change in dimensions take place only in one direction, that is when deformations in the other two directions are constrained so that the dimensions remain unchanged. These conditions occur in specimens or structures where two dimensions are much larger than the third [31,32].

When a λ/2π << 1, *i.e.*, when the extent of attenuation per wavelength is small, as in most practical applications, the following simplified formulas can be used to calculate the two components of the complex longitudinal modulus:

*L*′ = ρ*c*^2^; *L*″ = 2ρ *c*^3^*a*/ω (4)

where ω is the angular frequency (ω = 2π*f*).

## 3. Contact Ultrasonic Cure Monitoring

### 3.1. Experimental Set-Up for Contact Ultrasonic Cure Monitoring

**Figure 1:**
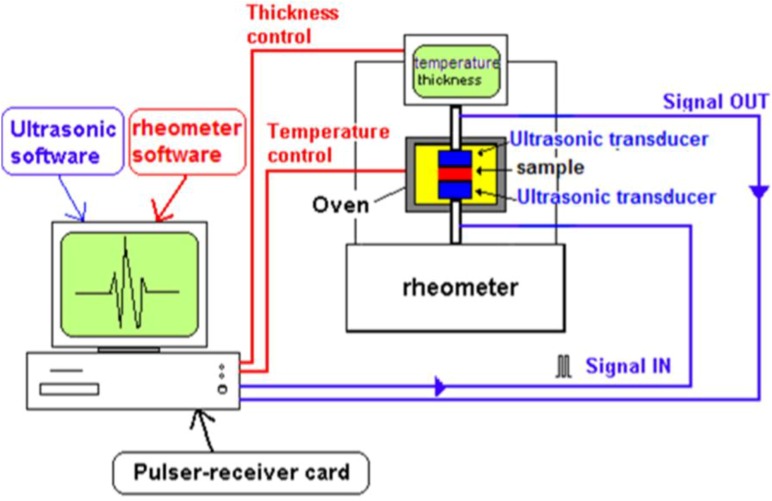
Schematic drawing of the ultrasonic set-up for cure monitoring (adapted from [18]).

A sketch of the experimental setup for contact ultrasonic cure monitoring is reported in Figure 1 [18]. It consists of two custom made ultrasonic probes able to propagate longitudinal waves in the 1–10 MHz range. The design and performance of ultrasonic probes have been recently optimized by finite element modeling [33]. The ultrasonic probes are fitted into the tools of a parallel plate rheometer and connected to a pulser-receiver system integrated in a personal computer. The pulser-receiver card generates a train of high-frequency electric impulses, inducing the vibration of the piezoceramic transducer inside the probe, and provides an analog/digital conversion of the signal. A dedicated software is used to sample and display on the monitor of the PC the electric signal resulting from the piezoelectric vibrations. The longitudinal velocity and attenuation are determined by comparing the peak time and peak amplitude of the reflected wave with those of a reference signal recorded without sample.

The measurements can be performed both in pulse-echo and transmission mode, with the sample loaded between two ultrasonic probes or one ultrasonic probe and a parallel plate of the rheometer, respectively. The set-up offers, thus, the additional advantage of being able to operate simultaneously as a parallel plate rheometer and as an ultrasonic dynamic mechanical analyzer [34].

The thickness of the polymer sample is accurately measured during the experiment by a device incorporated in the rheometer. This makes it possible to measure also any change in thickness by following the vertical movement of the upper transducer, which is driven by the rheometer movable stage. Moreover, the parallel plate of the rheometer enables the application of a constant contact pressure between sample and ultrasonic probe [35].

The sample can be subjected to temperature scans within the rheometer oven since the ultrasonic transducers can withstand higher temperatures (up to 200 °C) than the commercially available transducers used for non-destructive inspection. This allows broadening the application range of the ultrasonic dynamic mechanical analysis. The system has been successfully applied by the authors of this review to study the transitions in linear and crosslinked polymers and the curing of thermosetting resins, as can be presented in the application section.

### 3.2. Results Obtained by Contact Ultrasonic Cure Monitoring

#### 3.2.1. Phase Transformations During Cure: Gelation and Vitrification

The experimental work, carried out with the contact ultrasonic set-up realized in our laboratory, has been focused on evaluating the reliability of UDMA in monitoring the phase transformations that occur during the cure of thermosetting resins, called *gelation* and *vitrification*. Gelation is defined as the phase transformation during which the curing resin transforms from a viscous liquid to an elastic gel, the viscosity of the system becomes infinite and the shear response of the resin grows from zero to finite values. Once the resin has gelled, it cannot be further processed, and operations as fiber impregnation or void removal are no longer possible.

Vitrification is that phase transformation of a curing thermosetting resin corresponding to the formation of a glass solid. At vitrification, the glass transition temperature of the resin, continuously increasing during the cure, approaches the cure temperature. As a consequence, the mobility of the reactive groups is strongly limited, the polymerization becomes diffusion controlled and may continue very slowly. During composites manufacturing, at vitrification the curing is considered practically terminated, being possible to extract the composite from the mold or mandrel without damaging the component. Knowledge of vitrification times may be employed also to optimize cure cycle times. Therefore, from the perspective of a material processor, it is important to follow the progression of the reaction and identify the occurring of gelation and vitrification.

**Figure 2:**
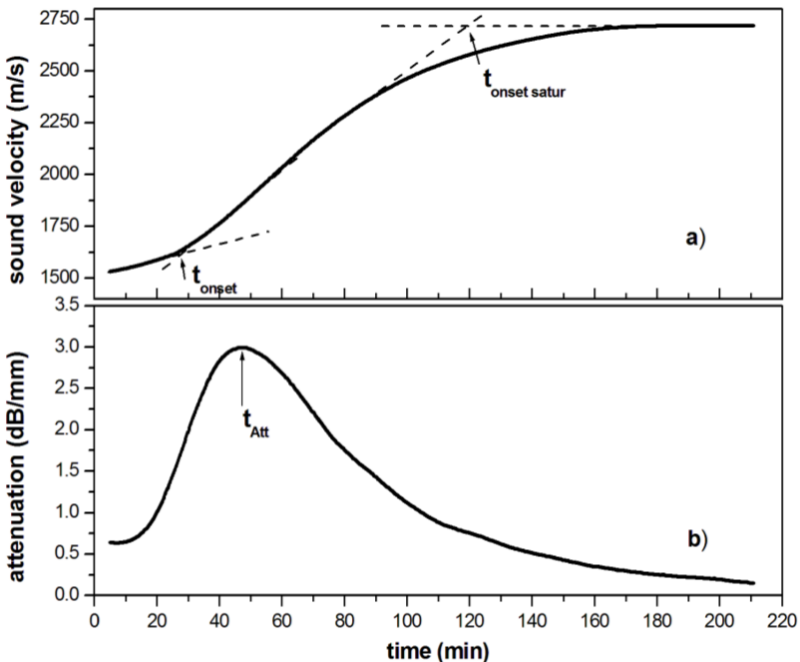
**(a)** Longitudinal velocity; and **(b)** attenuation measured at 2 MHz during the isothermal cure of an unsaturated polyester resin at 30 °C.

A typical curve of ultrasonic velocity *versus* cure time is reported in Figure 2a for the isothermal cure at 30 °C of unsaturated polyester resin (UPE) resin, catalyzed with methyl ethyl ketone peroxide (MEKP) as an initiator and cobalt octoate as a promoter, used at 1.5 and 0.5 parts per hundred parts of resin (phr), respectively. For the specified reacting system, the chosen curing temperature is in the region between gel *T*_g_ and *T*_g∞_. As reported by Enns and Gillham [36], gel *T*_g_ is defined as the temperature at which the gel time is equal to the time required for the vitrification, while *T*_g∞_ is the maximum glass transition temperature achievable by the resin when it is fully reacted. In this curing condition, gelation occurs before vitrification.

As observed in Figure 2a, the sound velocity curve has a sigmoid shape, characterized by three zones that can be associated with three different stages of the crosslinking reaction [18]. In the first stage of the cure, the velocity is nearly constant because the resin is still in the liquid state and has not yet developed a viscoelastic character. The second zone of the velocity curve starts after the gel point, when the ultrasound velocity increases rapidly due to the formation of microgel domains large enough to produce a measurable viscoelastic response to ultrasonic waves. The gel time can be determined from the onset of the velocity increase (*t*_onset_), as the crossover of two tangents drawn in the area of interest [37], as shown in Figure 2a.

The third zone of the velocity curve is characterized by a progressive decrease of the rate of change of the longitudinal velocity, which converges asymptotically to a plateau value. This zone corresponds to the vitrification stage, where a large increase in the crosslinking density with a concomitant reduction in chain mobility within the molecular network occurs. As a consequence, the crosslinking reaction becomes diffusion controlled resulting in a freezing of the network structure, whereby stable values for sound velocity are achieved. The cure time required until the sound velocity curve begins to saturate forming a plateau (*t*_onset satur_) can be defined by the crossing of two tangents drawn in the area of interest, as shown in Figure 2a. This value can be used as a reference point denoting the slowdown of the reaction as the *T*_g_ approaches maximum values achievable for the system under specified curing conditions, similarly to what happens in a dynamic mechanical experiment [38]. It should be kept in mind that the values of *t*_onset satur_ times are difficult to determine with high accuracy because their positions will vary depending on how the tangents are placed and should only be used as orientation values.

The overall velocity increment during the ultrasonic measurement is very high (about 1200 m/s), indicating that the transition from viscous liquid state to glassy solid state is accomplished by large changes in acoustic properties.

The curve of the ultrasonic attenuation *versus* cure time (Figure 2b) is bell-shaped with a distinct peak associated with α relaxations at the glass transition temperature (*T*_g_) [18,22]. The time in correspondence of the attenuation peak (*t*_Att_ in Figure 2b) can be used to identify the onset of vitrification, defined as the time at which the difference between the cure temperature and the *T*_g_ of the curing system, continuously increasing during the cure, becomes very small [39]. A peak is observed also in the measurements of the loss factor in dynamic mechanical analysis and in the dielectric analysis [26,31,40,41,42]. The attenuation peak is strongly anticipated with respect to the glass transition, defined in terms of free volume, being a dynamic mechanical response of the resin in the megahertz range. This can explain the reason why the cure still continues after the attenuation peak and why *t*_onset satur_ is far distanced from t_Att_.

As reported by Babayevsky and Gillham [41], who analyzed the shear dynamic–mechanical properties of an epoxy resin during the cure at low frequency (1 Hz), two peaks in the damping factor are expected during the cure of a thermosetting resin. The first one (weaker) has been attributed to gelation and the second (stronger) to the transition in the glassy state (vitrification). However these results are obtained in shear conditions, while the behavior of a material (when longitudinal ultrasonic waves are used) is dominated by the bulk modulus. The first weaker peak is probably masked by the stronger second attenuation peak associated to the vitrification process.

The experimental results above reported demonstrate that ultrasonic technique can identify the two critical stages of the crosslinking process. Since these irreversible changes occur at different rates and clearly affect the evolution of the resin mechanical properties, they can be easily detected by ultrasonic techniques [15,16]. Moreover, a relationship exists between the attenuation a and the dynamic viscosity η, allowing to differentiate the viscosity between two polymerization states [43,44].

**Figure 3:**
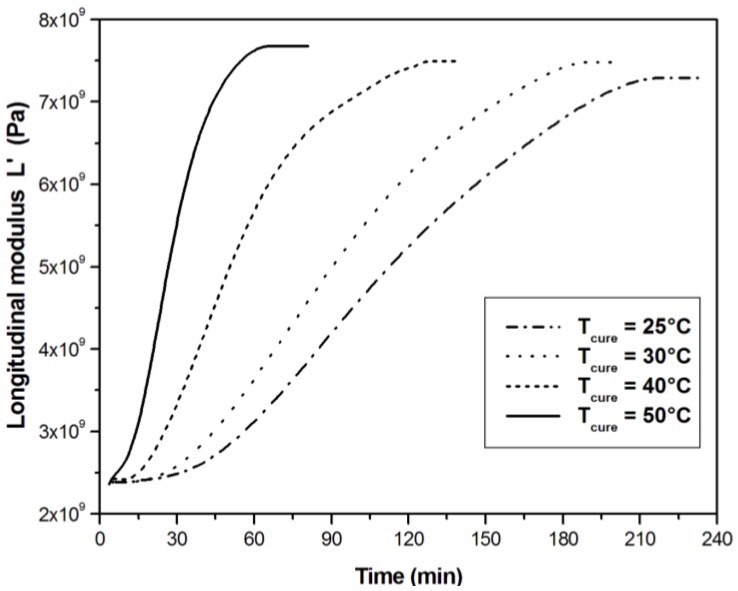
Longitudinal modulus *L*′ measured at 2 MHz during the isothermal cure of unsaturated polyester resin at different cure temperatures.

**Figure 4:**
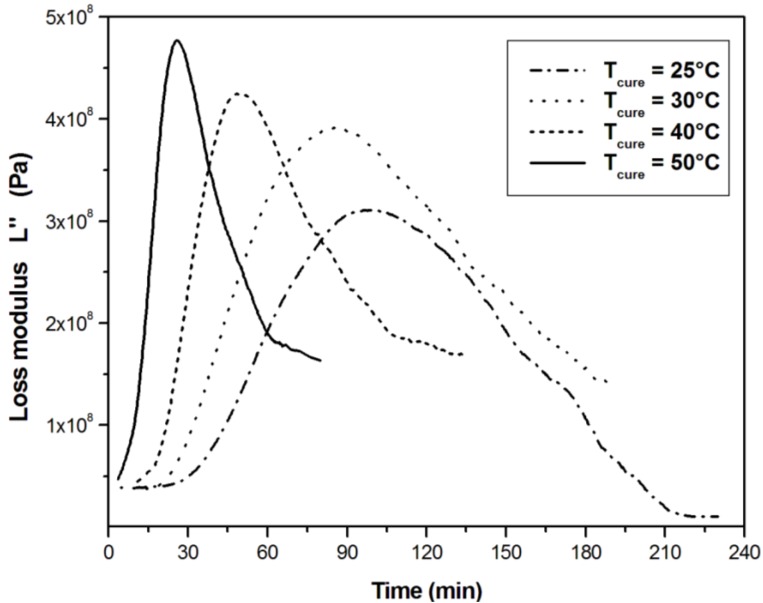
Loss modulus *L*″ measured at 2 MHz during the isothermal cure of unsaturated polyester resin at different cure temperatures.

In Figure 3 and Figure 4, the development of *L*′ and *L*″ with curing time is reported for sample of UPE resin cured at different temperatures. The longitudinal modulus *L*′ (Figure 3) follows a sigmoid curve, with the maximum increase after gelation, and reaches a stable value at the end of the cure with a typical behavior of an autocatalytic cure mechanism, which can be inferred by the rapid increase of the modulus after gelation. At the beginning of the cure, the longitudinal modulus is already high (2.4 GPa) because is dominated by the bulk component, being the shear modulus *G*′ of the liquid reactive system equal to zero. Only in correspondence of gelation, the shear component starts to contribute to the *L*′ increase as a consequence of the development of elastic properties given by the crosslinking reaction.

As the viscoelastic losses become maximum, the loss modulus *L*″ (Figure 4) presents a big peak, which falls rapidly as vitrification takes place and elastic response becomes significant. The ultrasonic data show a continuous development of mechanical properties after the reaction becomes diffusion controlled.

The effect of temperature is evident on the shape of the moduli curves reported in Figure 3 and Figure 4. The slope of the longitudinal modulus curves increases with the cure temperature in accordance with the cure kinetics. As the cure temperature increases, the peak of loss modulus shifts to the earlier stage of the cure reaction and becomes sharper.

**Figure 5:**
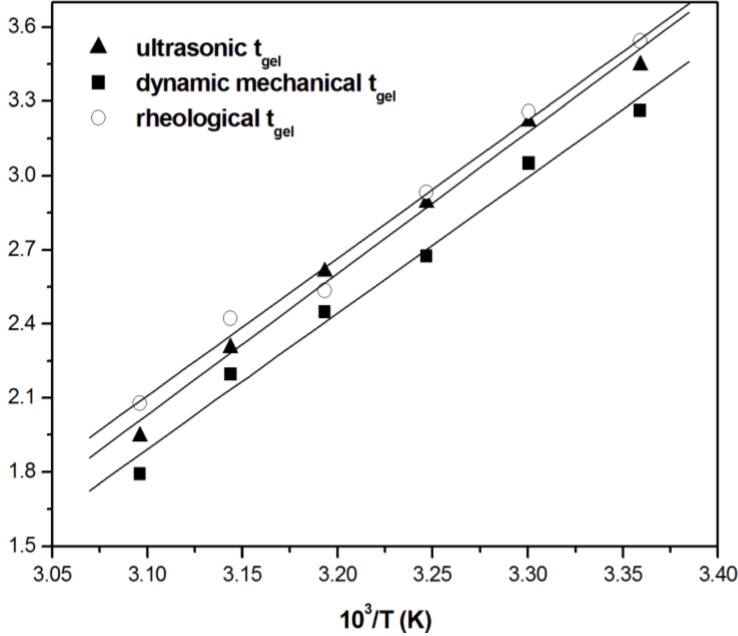
Arrhenius plot of the gel times calculated by different techniques *versus* the reciprocal of the isothermal cure temperature. Reprinted with permission from [45]. Copyright 2007 WILEY-VCH Verlag GmbH & Co.

The ultrasonic measurements can be used to calculate the activation energy of gelation reactions by means of *t*_onset_, which, as mentioned before, can be used as a measure of gel time. Being gelation a thermally activated process, the dependence of gel time, *t*_gel_, on the cure temperature can be described by an Arrhenius equation:

*t*_gel_ = *A* exp(−*E*_a_/*RT*) (5)

where *E*_a_ is the activation energy for the crosslinking reaction; *T* the absolute cure temperature; *A* the pre-exponential factor; and *R* the universal gas constant, which is equal to 8314 J/(mol K). The plot of ln *t*_gel_ (expressed in min) as a function of the reciprocal of absolute temperature (reported in Figure 5) can be used to calculate the activation energy from the slope of the curve.

The gel times obtained from ultrasonic cure monitoring are compared in Figure 5 with those obtained from steady rheology (at a constant shear rate of 0.5 s^−1^) and dynamic mechanical analysis (at 1 Hz), both performed on a parallel plate rheometer. Gel times from steady rheological measurements have been assumed to occur at the point at which viscosity reached a value of 1000 Pa·s [46,47]. Gel times from oscillatory measurements have been assumed to occur at the crossover of *G*′ and *G*″ curves [48]. Activation energies for the cure process of 43.8, 44.7 and 41.2 kJ/mol, respectively obtained from the rheological, dynamic mechanical and ultrasonic results, indicate a good agreement among the different techniques and the literature data (40–60 kJ/mol) [49] for polymerizations promoted by free radicals as in the case of unsaturated polyester resins.

#### 3.2.2. Ultrasonic Degree of Conversion and Sensitivity to Vitrification

To better highlight the sensitivity of ultrasound to cure monitoring, the results of the ultrasonic measurements have been compared with those obtained from Differential Scanning Calorimetry (DSC) on the basis of degrees of reaction obtained from curing experiments at the same temperature. In isothermal DSC experiments, assuming that the amount of heat generated through curing can be directly related to the conversion of the reactive groups, the degree of reaction α*_DSC_* is calculated as [50,51,52,53]:

(6) $$\alpha_{DSC}=\frac{\Delta H(t)}{\Delta H_{TOT}}=\frac{1}{\Delta H_{TOT}}\int_{0}^{t}\frac{dH}{dt}dt$$

where Δ*H*(*t*) represents the partial heat of reaction developed during an isothermal DSC experiment; and Δ*H*_TOT_ is the maximum heat of reaction measured in non-isothermal experiments, taken as a reference value.

An ultrasonic degree of reaction α_US_ can be obtained from ultrasonic measurements according to the following equation:

(7) $$\alpha_{US}=\frac{L\prime -L_{0}}{L_{\text{max}}^{\prime }-L_{0}^{\prime }}$$

where *L*′_0_ and *L*′_max_ are respectively the minimum and the maximum value of the longitudinal modulus.

**Figure 6:**
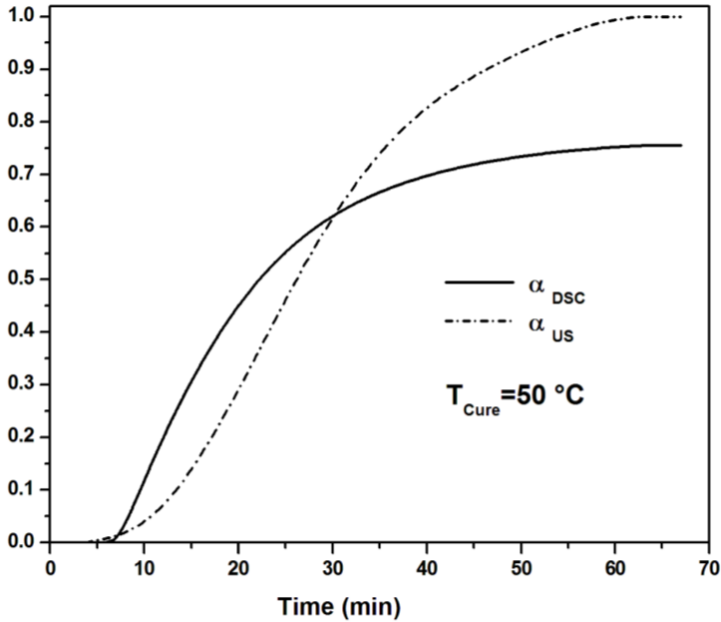
Normalized degree of reaction calculated from DSC and longitudinal modulus (measured at 2 MHz) for an UPE resin during the isothermal cure at 50 °C (adapted from [18]).

The comparison between the degrees of reaction from ultrasonic and calorimetric experiments in isothermal conditions is shown in Figure 6. At the beginning of the cure, the degree of reaction measured by DSC increases more rapidly than the ultrasonically measured value, since the mechanical properties of the resin before the gel point are scarcely affected by any increase in molecular weight. At gelation, the growing network gives rise to a significant increase of velocity. During the later stages of the cure, significant changes in the longitudinal velocity and modulus *L*′ occur, while the degree of cure is fully developed and remains nearly constant. There is a non-linear correlation, therefore, between longitudinal modulus and degree of cure.

Compared to DSC and dielectric analysis, techniques commonly used to monitor the curing of thermosetting resins, ultrasonic wave propagation appears to be more sensitive to changes in modulus occurring during the vitrification stage when the reactions become diffusion controlled. In this stage of the cure process, when the resin is almost crosslinked and in the state of a glassy polymer, very small changes in conversion, not distinguishable using DSC or dielectric analysis, will lead to an increase in crosslink density and modulus, still detectable by monitoring the sound velocity [16,17].

Moreover, an additional advantage of ultrasonic cure monitoring is its ability to monitor the temporal development of storage modulus over the whole curing reaction. For isothermal cure, even the newer rheological instruments require at least one change of plates (large plates for liquid to rubberlike state and small plates for the rubber to glass-like state) or at least two separate experiments per cure temperature before a change in modulus can be accurately measured throughout cure.

#### 3.2.3. Determination of Elastic Properties after Gelation

The ultrasonic technique can be used to evaluate the effect of the final network structure on the evolution of mechanical properties of the curing resin after gelation. To this aim, the statistical approach of Miller and Macosko [54,55], which correlates the crosslinking density of the resin to the degree of reaction, has been applied. The results, reported here, are based on studies of the cure of abifunctional epoxy resin, consisting of a diglycidylether of bisphenol A (DGEBA) with an epoxy equivalent weight of 184–190 g/mol. The resin has been mixed with tetrafunctional amines of different molecular weight, commercially known as Jeffamine D230 and D400.

The theory of Miller and Macosko enables to calculate the crosslinking density (μ) and the concentration of active network chains (ν) through the determination of the probability *P*($F_{A}^{out}$), that is the probability that the event $F_{A}^{out}$ occurs. For the studied system, $F_{A}^{out}$ is defined as the event after gelation when, taking any amine group and moving out from the molecules to which the group is connected, a dangling end is reached. It is evident that the probability $F_{A}^{out}$ can be defined only after gelation since, before gelation, an infinite network does not exist.

**Figure 7:**
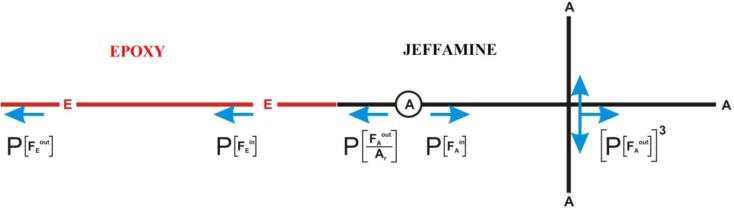
Scheme of the probability distribution.

The theory derives an expression which provides the probability *P*($F_{A}^{out}$) as a function of the amine conversion *p*_A_ For the studied epoxy system, the law of the probability has the following form:

(8) $$P\left(F_{A}^{out}\right)=P\left(F_{A}^{out}/A_{r}\right)p_{A}+\left(1-p_{A}\right)$$

where $P\left(F_{A}^{out}/A_{r}\right)$ is the probability to reach a functional amine group not connected to the network, when one or more remaining functional groups of the monomer have reacted. In Figure 7a scheme of the probability function is reported, taking into account that the functionality of the epoxy resin and amines used in this work are 2 and 4, respectively.

Equation 8 can be simplified by considering that if the reactive group A has reacted, the probability to find a free end by moving out from A coincides with the probability to find a free end by moving towards the inner of epoxy monomer or the outer of epoxy monomer, as reported in Figure 7, that is

(9) $$P\left(F_{A}^{out}/A_{r}\right)=P\left(F_{E}^{in}\right)=P\left(F_{E}^{out}\right)$$

At this point, by reiterating the total probability of finding a free end in the amine monomer for the epoxy monomer, it is possible to write:

(10) $$P\left(F_{E}^{out}\right)=P\left(F_{E}^{out}/E_{r}\right)\times p_{E}+\left(1-p_{E}\right)$$

Since

(11) $$p_{E}=rp_{A}$$

and

(12) $$P\left(F_{E}^{out}/E_{r}\right)=P\left(F_{A}^{in}\right)=P^{3}\left(F_{A}^{out}\right)$$

Equation 8 can be written in the following form:

(13) $$rP^{3}\left(F_{A}^{out}\right)p_{A}^{2}-P\left(F_{A}^{out}\right)-rp_{A}^{2}+1=0$$

here *r* is the stoichiometric ratio; and *p*_A_ is the amine conversion. Since *r* is equal to 1 in the studied formulation and *p*_A_ can be assumed equal to the degree of reaction (α_DSC_) calculated from calorimetric experiments, Equation 13 becomes:

(14) $$\alpha =\sqrt{\frac{P\left(F_{A}^{out}\right)-1}{P^{3}\left(F_{A}^{out}\right)-1}}$$

At this point *P*($F_{A}^{out}$) can be obtained as a function of the degree of conversion of amine groups, by polynomial fitting. In particular, for postgel range of *p*_A_ (*p*_A_ > 0.57) the following polynomial expression is obtained:

(15) $$P(F_{A}^{out})=c_{0}+c_{1}\alpha +c_{2}\alpha^{2}+c_{3}\alpha^{3}+c_{4}\alpha^{4}+c_{5}\alpha^{5}$$

where: *c*_0_ = 8.076; *c*_1_ = −30.954; *c*_2_ = 58.083; *c*_3_ = −62.135; *c*_4_ = 35.455; and *c*_5_ = −8.524.

Once *P*($F_{A}^{out}$) has been calculated, the probability that a tetrafunctional amine molecule, as the Jeffamine used in this study is connected to m segments, belonging to the infinite network, is:

(16) $$P(X_{m,4})=\left(\begin{array}{c} 4\\ m \end{array}\right)P{\left(F_{A}^{out}\right)}^{4-m}{\left[1-P(F_{A}^{out})\right]}^{m}$$

An amine molecule is an effective crosslinking point only if *m* > 3. The crosslinking density μ is therefore given by

(17) $$\mu =\left[A_{0}\right]\sum_{m=3}^{4}P(X_{m,4})$$

where [A_0_] represents the initial concentration of amine, equal to 1.16 × 10^–3^ mol/cm^3^ for D230 and 9.57 × 10^−4^ mol/cm^3^ for D400. Since at each junction point can be associated *m*/2 connected chain segments, the density of active network chains, ν, is obtained as:

(18) $$\nu =\left[A_{0}\right]\sum_{m=3}^{4}\frac{m}{2}P(X_{m,4})$$

**Figure 8:**
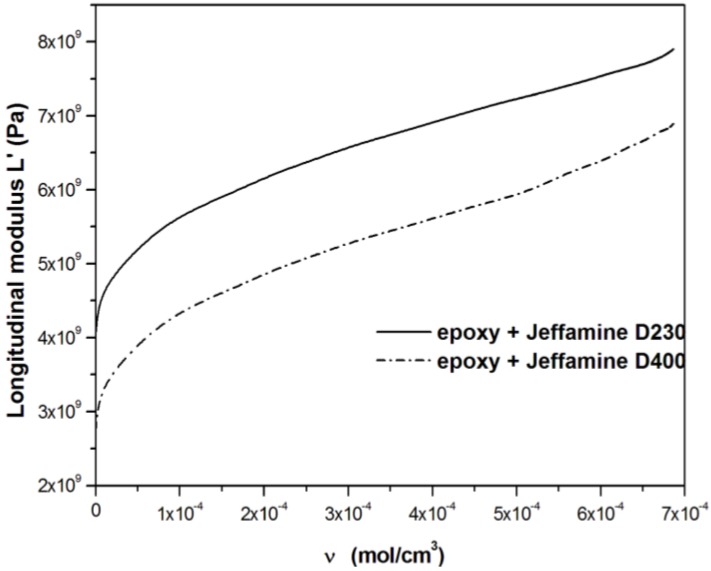
Correlation between *L*′ and the concentration of active network chains ν after the gel point, during the cure of epoxy resin DGEBA at 85 °C with Jeffamine D230 and D400.

Therefore, it is possible to obtain a plot of the concentration of active network chains, ν, *versus* the degree of reaction. The density of active network chains, ν, is an important parameter since it is proportional to the elastic energy stored by the curing resin and the mechanical properties of the curing gel depend on it. It is therefore possible to correlate ν to the longitudinal modulus *L*′, obtained from ultrasonic measurements, which is a measure of the stored elastic energy, as shown in Figure 8.

A large part of the curve is linear as predicted by the theory of rubber elasticity for the shear modulus [56,57]:

*G*_0_ = ν*RT* (19)

where *G*_0_ is the equilibrium shear modulus; *R* the constant for ideal gas; and *T* the absolute temperature. Since shear, Young and bulk moduli are correlated, also the longitudinal modulus *L*, function of shear and bulk moduli as reported in Equation2, should be characterized by a linear dependence on ν.

Although, the theory of rubber elasticity predicts moduli far too small for extremely high crosslinked polymers, a linear correlation between *L*′ and ν is observed during crossinking, as also observed by Nielsen [56].An initial deviation from linearity is observed close to the gel point where the rate of increase of *L*′ is higher than that of ν. A further deviation from linearity is observed at the end of the crosslinking reaction for the system with D400 amine. Since the maximum degree of reaction, measured by calorimetric analysis, is lower than 1 (precisely 0.8), it means that vitrification occurs during reaction leading to a diffusion controlled reaction termination. Therefore, this transition to the glassy state could be responsible of the fast non-linear increase of *L*′ with ν.

#### 3.2.4. Post-Cure Monitoring

**Figure 9:**
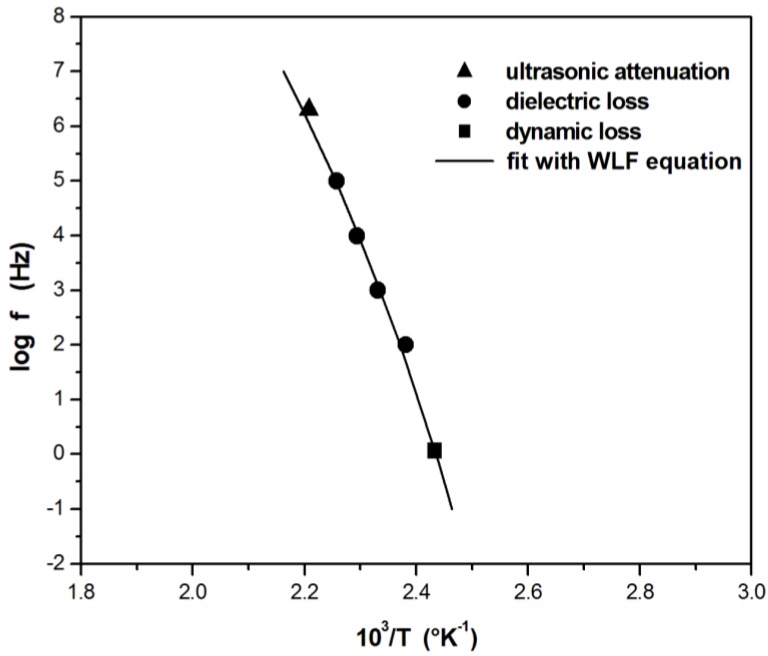
Frequency dependence of the relaxation peaks of unsaturated polyester networks as measured by DMA (square), DEA (circles) and UDMA (triangle). The data are fitted by the WLF equation (continuous line). Reprinted with permission from [8]. Copyright 2005 Wiley Periodicals, Inc.

Ultrasonic wave propagation can be used also to monitor isothermal and non-isothermal post-cure cycles during manufacturing of composite materials and to shorten the procedure of finding optimal post-curing conditions [7,19]. During a post-cure cycle of thermosetting resins, the residual reactive groups, immobilized in the glassy state byvitrification during a previously carried out isothermal cure step, gain molecular mobility, which promote the formation of additional crosslinks. After the post-cure, the reaction can be complete, and the maximum achievable glass-transition temperature can be reached. The ultrasonic technique is able to get a better insight of the relaxation phenomena [58,59] and, if coupled with low frequency DMA, the behavior in a wide frequency range can be investigated. For example, ultrasonic wave propagation, dynamic mechanical analysis, and dielectric analysis have been used to monitor relaxation phenomena during the non-isothermal post-cure of unsaturated polyester resin [8]. The measurements have covered six decades of frequency. The frequency and temperature dependence of the main α relaxation has been evaluated. It has been found that, despite the great difference in frequency spanning from 1 Hz for DMA to several MHz for UDMA, the data on highly crosslinked polymers can be fitted by the Williams-Landel-Ferry equation by plotting the logarithm of the frequency of the loss factor (*L*″/*L*′) against the reciprocal of the peak temperature (*T*_g_) (see Figure 9). The Williams-Landel-Ferry equation [60], has the following expression:

(20) $$log\frac{f}{f_{0}}=\frac{-C_{1}\times (T-T_{0})}{C_{2}+(T-T_{0})}$$

where *f*_0_ and *T*_0_ are the reference frequency (1 Hz) and reference temperature; which in the present case is the *T*_g_ measured at 1 Hz; C_1_ and C_2_ are constants, initially speculated as having universal values, but actually depending on the material system and on the property measured [61]. The constants *C*_1_ and *C*_2_, obtained by a nonlinear fitting procedure, have values of 22 and 111.9 K, respectively.

## 4. Air-Coupled Ultrasonic Cure Monitoring

Despite the potential advantages, the diffusion of ultrasonic cure monitoring in industrial applications is hampered by the necessity of resin-transducer contact in order to propagate acoustic waves into the curing composite without significant losses. At the interface between two materials, only a portion of the sound is transmitted depending on how close are the acoustic impedances of the two materials. Similarly, at the air-solid interface, only a very small fraction of the sound energy is transmitted since air has a very low acoustic impedance. For this reason, the ultrasonic inspection is usually carried out by means of a fluid coupling agent between the ultrasonic transducer and the material under investigation. Nevertheless, the resin-transducer contact is not always possible in some manufacturing technologies and the fluid used as a coupling medium could adversely affect the resin curing or the other component of the composite material, for example wood [62,63].

More recently, our research has been addressed to air-coupled ultrasound to overcome the above-mentioned limitation of the resin-transducer contact. Air-coupled ultrasound has been recently enabled by the availability on the market of a new kind of ultrasonic transducers, called air-coupled ultrasonic transducers [64,65,66,67]. They minimize the impedance mismatch between air and transducer, thereby providing a good acoustic transmission also through the air gap. We have proposed air-coupled ultrasonic cure monitoring as a very promising monitoring technique with a great potential for some open mould manufacturing processes, such as filament winding.

### 4.1. Experimental Set-Up for Air-Coupled Ultrasonic Cure Monitoring

**Figure 10:**
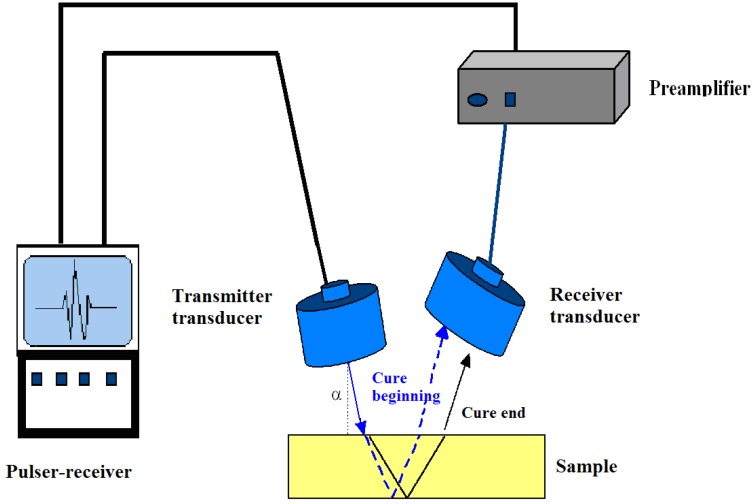
Experimental set-up for the generation and detection of longitudinal waves by air-coupled ultrasound in the pitch-catch mode (adapted from [45,68]).

In our laboratory an experimental setup has been developed for propagating compression waves during the overall curing reaction. Two non-focused air-coupled ultrasonic transducers of 500 KHz nominal frequency are connected to a pulser-receiver device and placed on the same side of the test material, in the so-called pitch-catch configuration (Figure 10) [45,68].

A large experimental and theoretical work has been necessary to increase the signal-to-noise ratio, through the optimization of the geometry set-up in terms of positioning angles of probes, distance between the air-contact probes, air distance between probe and sample, and active area of the receiver transducer. It has been taken into account that, during the curing, the sound velocity of the curing system is increasing due to the very large increase in the elastic modulus, thus changing continuously the path of the acoustic air in the specimen, as reported in Figure 9.

Another feature of air-coupled cure monitoring is the heating of the air between transducer and sample, due to the exothermic character of curing reaction. It is known that the air velocity increases of 2.8 m/s for an increase of 5 °C of air temperature. This effect has been taken into account by sampling the air velocity during the experiment and correcting the measured value of time of flight with the corresponding value in air, which may changes significantly during the cure.

### 4.2. Results Obtained by Air-Coupled Ultrasonic Cure Monitoring

**Figure 11:**
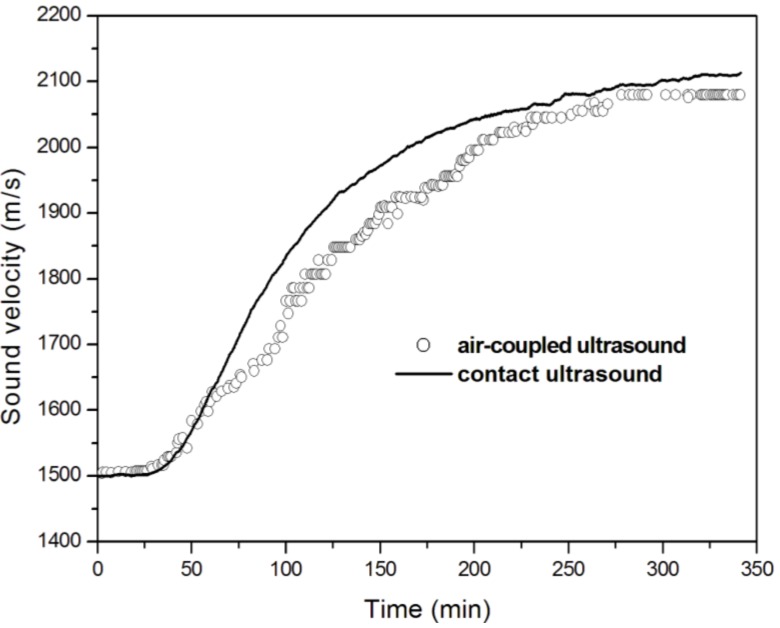
Comparison of longitudinal velocity changes, obtained both by air-coupled (0.5 MHz) and contact (2 MHz) transmission mode, during the cure at room temperature of an unsaturated polyester resin (adapted from [68]).

The progress of polymerization of an unsaturated polyester resin has been monitored through the variation of the time of flight of the propagating longitudinal waves. The air-coupled ultrasonic results have been compared in Figure 11 with those obtained from conventional contact ultrasonic measurements. The velocity curves obtained by the two ultrasonic techniques are very similar, with the three zones before described or contact UDMA. This demonstrates the reliability of air-coupled ultrasound in monitoring the changes of viscoelastic properties at gelation and vitrification.

The effect of cure temperature and cure promoter on the cure kinetics has been studied. Both the shapes of the velocity and attenuation curves are strongly affected by the amount of curing agent present in the resin, in accordance with the kinetics of the reaction.

The position of the transducers on the same side of the sample makes this technique suitable for on-line cure monitoring during several composite manufacturing technologies.

## 5. Conclusions

In this paper, the results of ultrasonic cure monitoring of thermosetting resins, obtained by the author’s research group in the last decade, have been reviewed. Two kinds of ultrasonic techniques are presented: contact ultrasound, which is the conventional technique using a fluid coupling medium between transducer and sample, and air-coupled ultrasound, *i.e.*, without any contact between the ultrasonic transducer and the material under test. Suitable experimental set-ups, developed for generation and detection of ultrasound and for data analysis, have been described.

Ultrasonic wave propagation has been used to characterize the phase transitions during the isothermal cure of different thermosetting resins by monitoring the time-evolution of ultrasonic longitudinal modulus and chemical conversion. The comparison of the ultrasonic properties with those of other well-assessed techniques (calorimetry, rheology, low frequency dynamic mechanical analysis) has indicated that gelation occurs at the onset of the velocity curve while the peak of attenuation is attributed to vitrification. Finally, the sensitivities of ultrasonic velocity to the last part of the cure has been also remarked upon.

The main advantages of the ultrasonic cure monitoring are: (a) a simple sample geometry and low cost; (b) the absence of force transducers and their sensitivity limitations at the end of reaction, when the sample is completely hardened; (c) the possibility of detecting the occurrence of gelation and monitoring the vitrification of resins in manufacturing processes; and (d) the potential for online and *in situ* monitoring during polymer and polymer matrix composite processing. Moreover, the possibility to eliminate the physical contact between the ultrasonic transducers and the resin can extend the application of the ultrasonic wave propagation to several technologies of composite material manufacturing.
